# Reciprocal effects between post-migration risk factors for mental health and barriers to access to treatment among refugees and asylum seekers: what have we learnt?

**DOI:** 10.3389/fpsyt.2025.1725787

**Published:** 2025-12-04

**Authors:** Javier Bartolomei, Aymeric Reyre

**Affiliations:** 1 Department of Psychiatry, Geneva University Hospitals, Geneva, Switzerland; 2 CESP-INSERM, U1018, Developmental Psychiatry Team, Paris-Saclay University, UVSQ, Villejuif, France

**Keywords:** asylum seekers, barriers to treatment, post-migration risk factors, mental health policies, circular reinforcement

## Abstract

The last decade has been marked by increasing numbers of forcibly displaced persons around the world, bringing new challenges for Western general and mental health services. For years, research has been focused mainly on pre- and per-migration risk factors for refugees and asylum seekers’ (RAS) mental health. Lately more attention has been given to post-migration risk factors whose impact seem to have been largely underestimated. Uncertain administrative processes, separation from family remaining in the home country, housing conditions, access to professional activity or training and social isolation appear to be associated with a higher prevalence of mental health disorders among RAS. In parallel, the organization of western medical services seem to be maladjusted to RAS’ needs and therefore contributes to post-migration stress factors impact on mental health. Integration of legal advice and job coaching in medical units, planned strategies to decrease social isolation, access to translators and cultural mediation in addition to access to training to local language are insufficiently considered. From our point of view, insufficient attention has been paid to the reciprocal effects between x and y. In order to better tailor healthcare provision to RAS, it seems necessary to take into account the mutual reinforcement of these issues and to develop approaches that are better suited to the complexity of the RAS experience and needs.

## 1 Introduction

The last decade has been marked by increasing numbers of forcibly displaced people around the world. In 2024, the United Nations High Commission for Refugees (UNHCR) estimated that their number could globally exceed 123 million. Among them, approximately 45 million are estimated to be refugees and asylum seekers (RAS) displaced across international boundaries (1, 2). The prevalence of post-traumatic syndrome and depressive disorder (the two groups of diagnosis that have been the focus of attention these last decades, while less is known about addiction or psychotic disorders), is known to be markedly higher than in the non-refugee population (3).

Different risk factors are known today to precipitate the emergence of psychopathology, among which there are pre-migration factors (traumatic factors in the country of origin), per-migration factors (what happens during the migratory journey) and finally post-migration risk factors (stress factors in the resettlement country). Until the year 2000, the first group of factors concentrated most of the attention in the specialized literature. Since then, various major studies (4, 5) have pointed out the significant impact of “post-migration living difficulties” on the clinical evolution of this population: uncertain administrative processes, permanent separation from family remaining in the home country, housing conditions, access to professional activity or training and social isolation are associated with a high prevalence of mental health disorders.

In addition, numerous specific barriers to access to mental healthcare for this particularly vulnerable population are now well known and are a major challenge to public mental health systems in Western European countries. Some of them are mostly internal (cultural understanding of psychiatric disorders, phenomena of self-stigmatisation, lack of trust towards public institutions) and others are external (information and geographical access to psychiatric outpatient units) and represent a kind of maladjustement to asylum’seekers needs that could increase the impact of post-migration risk factors on mental health.

From our perspective, we have to consider a mutual reinforcement between post-migration risk factors for RAS mental health and barriers to access to treatment that implies a holistic approach. Both should be taken into account simultaneously time to better our mental health system accuracy.

## 2 Post-migration risk factors

Around the year 2000, numerous studies demonstrated that resettling in a safe country was not a guarantee for improving psychological well-being (6, 7). The concept of post-migration living difficulties has progressively shed light on an astonishing variety of post-migration factors that have a real impact on the mental health of RAS (8, 9). Aragona et al. found that more than 70% of RAS included in their study had been confronted with post-migration risk factors, ranging from severe to very severe (7).

Asylum seeking procedur**es** are definitely among the most important factors (10). Laban et al. demonstrated that extended asylum-seeking procedures were associated with a higher prevalence of psychiatric disorders (major depressive disorder (MDD), anxiety and somatoform disorders) and had a considerable impact on quality of life, disability and physical health (4). In a longitudinal study, Silove et al. also pointed out how a favourable decision for refugee status could make a significant difference in recovery from trauma-related psychiatric symptoms (11). Subjects with post-traumatic syndromes have a specific need to feel safe: any factor compromising the feeling of security is likely to reactivate or amplify post-traumatic symptoms (nightmares, flashbacks, avoidance behaviours, hyperarousal, isolation). As a consequence waiting time is today considered as a significant risk factor that has a major impact on quality of life, and depressive and somatoform disorders (4, 5, 8, 9). In a recent systematic review, the length of the process of asylum application and the duration of stay were the most often cited difficulties in 9/22 studies (6).Separation from the family remaining in the home country can be a major risk factor for MDD and anxiety disorders (12, 13) for different reasons. Refugees and asylum-seeking patients commonly fear that their home country will take revenge on them by mistreating their families and friends, and that being safe implies putting their families in danger. This generates a real dilemma and intrapsychic conflict. Worrying about the lack of financial resources among their families and friends is another major source of anxiety for asylum-seeking patients (14). In a cross-sectional study exploring the association between pre- and post-migration stress factors among 2399 migrants holding a permanent visa, Chen et al. reported that 31% presented PTSD, and that 49% were deeply worried about their families (15). Separation from the family contributes to loneliness, social isolation and in case of lengthy asylum procedures to feelings of powerlessness when faced with negative events occurring in the home country: serious illness of a parent or a child, financial difficulties, etc. Moreover, being involved in asylum procedures does not allow RAS to invite their families to join them in the host country.

As mentioned previously, being unable to access work constitutes a major stress factor (16). A professional activity increases self-esteem and provides a social identity, which is of particular importance for people who have lost their past social identity in their home country. Professional activities favour a process of acculturation and social integration and trigger feelings of usefulness, in particular by way of the related financial resources, which they can send to the family back home. However, access to work permits for asylum-seekers differs greatly according to the host country’s integration policies and remains a major issue for the coming decades.

Housing problems are one expression of this difficulty in offering decent living conditions to this population (17). Among the unmet needs commonly cited by this population are: limited staff availability, reduced living space with no privacy, poor sanitation or kitchen amenities, and living in a place distant from the town centre without easy access to public transport or religious venues (18). In a study by Barberi et al., living in large centres (over 1000 inhabitants) was a significant predictor of pervasive and intense PTSD symptoms class (19).

The association between MDD and social isolation among RAS (20) was a major discovery in the late 90s. Lack of social support and poor social integration (15) progressively became points of interest. Feelings of rejection by the host community produce particular social and mental pain. Conversely, having friends is a protective factor for mental health (21) and social participation. Trust in others is known to have a protective effect for mental health when asylum-seekers are confronted with discrimination (22). The ability of a host country to propose a reliable model of integration to asylum-seekers remains a significant determinant of their mental health. Inversely racism (23) and repeated microagressions (24) are known to increase the impact of pre-migration factors and the prevalence of mental health disorders (16).

From a general perspective, interaction between mental health and poverty has been the focus of many researches for the general population (25). RAS arrive in societies where income inequality has already different consequences upon access to general and mental health services. The lack of access to a professional activity or training represents a consistent restriction of access to financial resources and therefore to health services particularly when insurance coverage is insufficient (26).

Some subgroups of asylum-seeking population have to be more specifically considered. In their recent work on Syrian women in Toronto, Sara de Sa et al. reported that there were specific post-migration risk factors for female refugees (27). The first concerns the risk of having sustained abuse because of gender or sexual orientation before or during the journey and not feeling able to talk about it for fear of being rejected by their own community. In addition, because women shoulder responsibility for childcare and education, access to personal care can be difficult. Recent studies have shown a kind of gender hierarchy, which reinforces male domination and control over women’s mental and physical health, and thus their access to health services. Interventions to reduce negative perceptions of psychiatric services and to foster help-seeking behaviours, especially for women, are among the present authors’ main recommendations. Support from spouses and family members (including those in the home country) and from the community is known to improve autonomy and access to mental healthcare. Psychoeducation (educating spouses and family members about symptoms) to increase knowledge of mental illness is thus a major therapeutic tool.

## 3 Barriers to access to treatment

According to Due’s definition, access to care can be described as “the relationship between those who seek to access healthcare and the healthcare resources available for them” (28). One question however remains: why are so many asylum-seekers and refugees with severe psychiatric disorders not treated in countries where efficient public psychiatric services exist (29, 30)? While globally, access to general healthcare for this population remains more or less comparable to that of the general population, access to psychiatric services remains remarkably low in high-income countries (28). In a study on access to mental healthcare for asylum-seekers and refugees, Boettchers et al. noted that 54.8% of their subjects presented psychological distress and only 28.9% of them perceived the need for psychotherapy (30). Further to this, having received mental healthcare increases the probability of social integration in the host country (31). Consequently, there are major public mental health benefits in detecting and treating asylum-seekers with psychiatric disorders as soon as possible.

Globally, we can distinguish two types of barriers to access to mental healthcare for asylum-seekers and refugees:

### 3.1 Cultural and personal barriers

This refers to everything that is linked to the person him or herself, their original culture and religion, their perceptions of public institutions in general, their representations of mental health and psychiatry, and what it means for their self-esteem to seek help for psychological reasons. Another aspect is how they anticipate reactions from of their community about the fact that they are seeking help.

Among main cultural and personal barriers, we have to mention:

Self-stigma is a well-known phenomenon in transcultural psychiatry, preventing psychologically distressed patients from seeking help and leading them to exclude themselves (30). Understanding a mental disorder as sign of weakness or a punishment from God triggers feelings of shame and self-exclusion behaviours (32). The influence of religious faith, the denial of mental illness, cultural gaps between patients and healthcare professionals and the mistrust of psychiatric institutions are regularly cited as risk factors for self-stigma behaviours and the consequent underuse of mental health services (33–35).The cultural acceptability of any psychiatric intervention needs to be explored, given that acculturation has a globally positive effect on acceptance (36, 37).

Fear of stigma from the person’s own cultural group or fear of disapproval on ethnic grounds is another expression of stigma which relates to the fear of being rejected by their own community, as mental illness is perceived as a kind misconduct or evidence of past shameful behaviours (38, 39). Denial of mental illness is commonly reported, and somatic symptoms are considered by the community to be much more acceptable (38). Indeed, help is supposed to be provided by the community and its figures of authority (religious or clan leaders) and not by external sources of help in some cultural references (39).

Lack of trust remains a persistent barrier to access mental healthcare, and it is frequently underestimated (40). Experiences of brutal psychiatric treatments in the past in the home country, or of racist discriminatory attitudes in the medical sphere in the host country could explain why psychiatric services are so suspect (33). Concerns about confidentiality associated with the fear of endangering family members still living in the home country are also mentioned, as well as fear of a negative impact on asylum-seeking administrative processes (40). Thus, a therapeutic alliance is essential to enable a cautious and precise exploration of past psychiatric experiences for the patients and/or their family members, and their beliefs about the links thought to exist between psychiatric services and immigration services.

### 3.2 Structural barriers

This term refers to different obstacles which, depending on the host country, prevent RAS from receiving specific psychiatric interventions from the very first day of their arrival.

The first is the lack of adapted, validated screening tools as there is a global consensus on the need to use culturally-validated questionnaires in order to improve the quality of psychiatric screening (41, 42). The inadequacy of the tools used can lead to underdiagnosis and delays in diagnosis of mental health disorders. It may also prevent nosographic discussion and the design of tailored therapeutic approaches. Duration of untreated symptoms has a significant impact on the prevalence and intensity of psychiatric symptoms (42). In their work, Hollifield et al. recalled for instance the use of the Refugee Health Screen 13- or 15-item questionnaire as an interesting tool to explore, arguing for its easy and quick use even for caregivers in somatic medicine (43).

Host language acquisition has lately been considered more and more as an essential skill, which not only plays a crucial role in the acculturation process, but has a significant impact on the mental health of this population (44). The use of interpreters should be considered as a basic need, mostly in the first months of the stay in the host country when language skills are still only basic (28). Recruiting family members to translate considerably limits confidentiality and freedom of expression.

The lack of a specific psychotherapeutic approach for PTSD and MDD has been pinpointed in different studies, with a marked tendency to offer non-specific pharmacological solutions, the efficacy of which remains unclear (45). In their work, Fuhrer et al. surprisingly observed a high proportion of asylum-seekers treated by psychiatrists without taking known guidelines about psychotherapeutic approaches to PTSD into account (42). The lack of trauma-related care (focused on the specific needs of a particular community) resulting from inadequate comprehensive transcultural training for caregivers probably aggravates this phenomenon (28).

Lack of RAS persons’ education and information about mental health disorders and about existing resources to treat them has been regularly pointed to in different studies as navigation in the mental health system of the host country can reveal itself extremely difficult. Misinformation about psychiatric disorders is therefore considered a major barrier to access to treatment (33).

Geographical distance to mental health unit, access to public transport, lack of financial resources access to child custody for women and delays before a first appointment (21, 46) are very concrete elements that can prevent access to mental health care. Delays before diagnosis are associated with lower remission rates for Post Traumatic Stress Syndrome, a deteriorated therapeutic alliance and an acculturation process of poorer quality with marked phenomena of social isolation (31, 47).

## 4 Reciprocal effects between barriers to access to treatment and post-migration risk factor

In today’s social and political climate, which is not particularly favorable to welcoming forcibly displaced persons into our Western communities, it is important to remember that improving the quality of care for these individuals is a moral, economic, and political necessity. While humanitarian arguments are less acceptable at present, it should be emphasized that this quality of care is important not only for controlling healthcare costs but also for enabling society to benefit from the contribution of RAS to work and social cohesion.

In our view, focusing the debate on the quality of care for RAS on removing barriers to care does not address the complexity of the determinants of mental health in this population. To accelerate progress in the quality of care for RAS, we believe it is necessary to take an integrated approach to both the issues of post-migration risk factors and barriers to access to care. There are many reciprocal interactions between these two categories, and we want to emphasize that they can reinforce each other.

**Figure 1:**
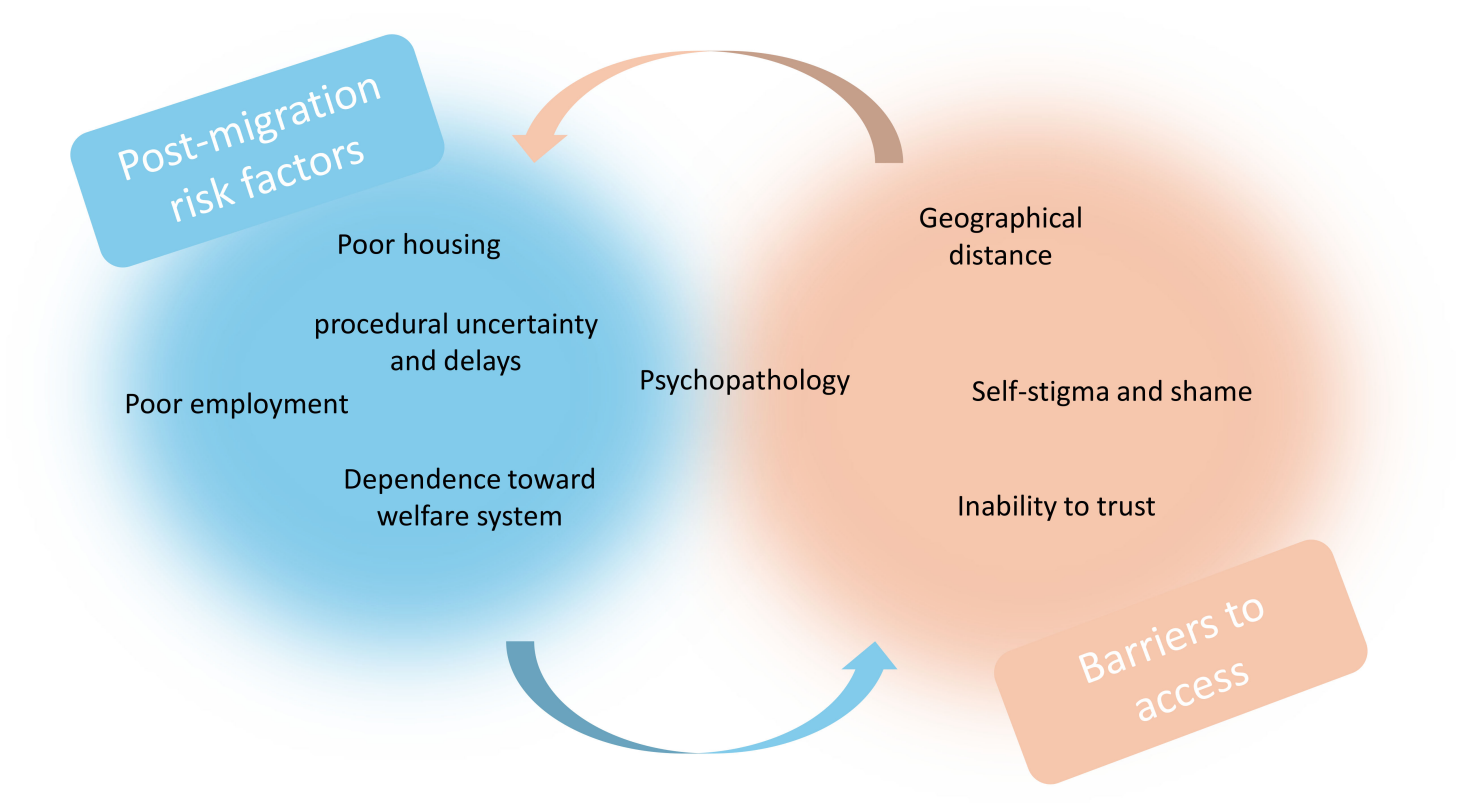
Mutual reinforcement between post-migration risk factors and barriers to accessing healthcare – example 1.

**Figure 2:**
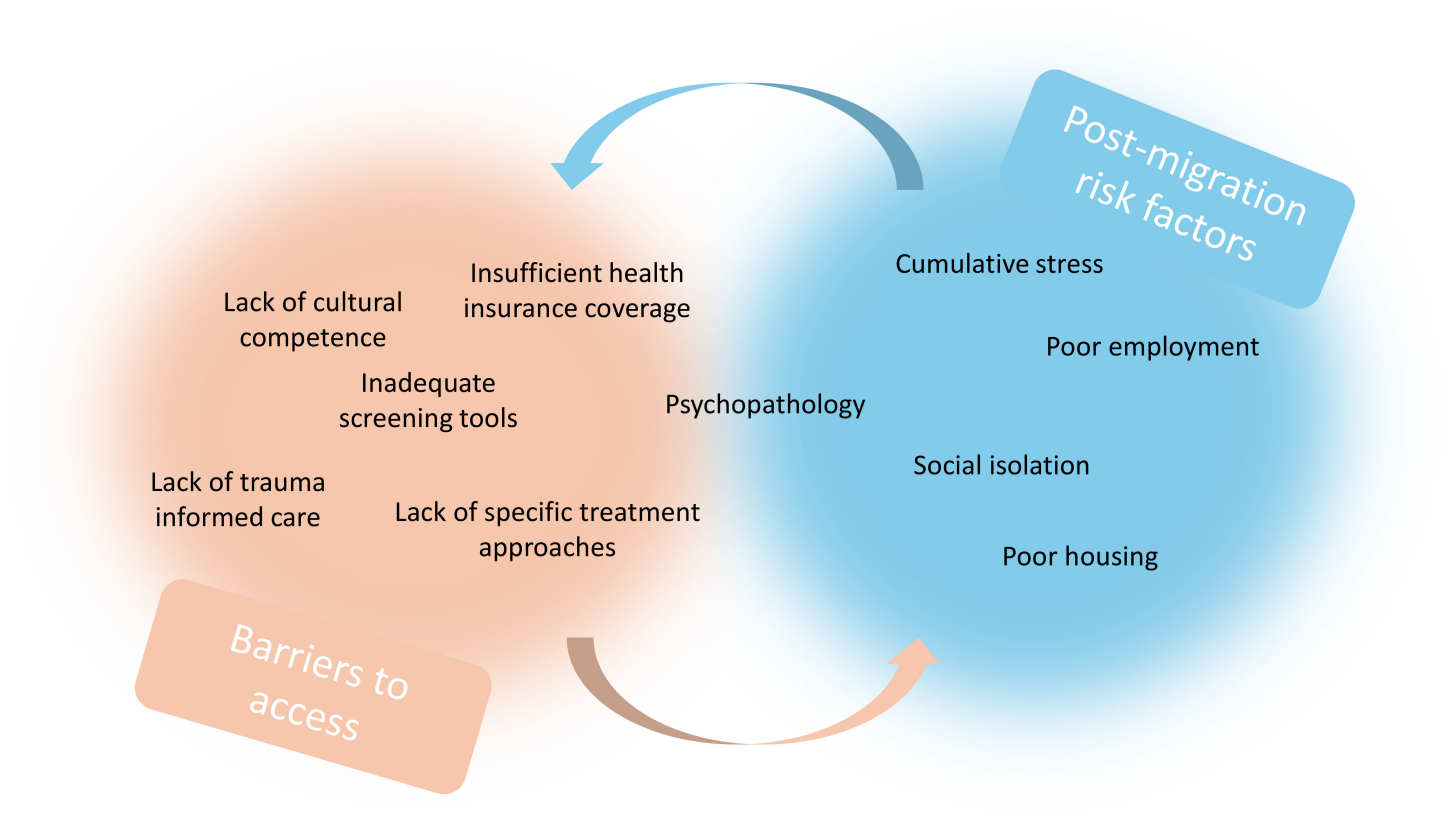
Mutual reinforcement between post-migration risk factors and barriers to accessing healthcare – example 2.

From our perspective, post-migration risk factors increase the probability of an emerging psychiatric disorder meanwhile barriers to access to mental health potentially increase the risk of the absence of an appropriate treatment of this disorder. To illustrate this mutual reinforcement between post-migration risk factors and barriers to accessing healthcare, we can examine the impact of procedures on the one hand and that of inappropriate screening tools on the other. Asylum procedures have several potential iatrogenic consequences (48). Difficulties in disclosing, notably for victims of sexual violence (49), have been reported implying a risk of dissociative episodes and retraumatization process, resulting in an increase in PTSD symptomatology. Uncertainty and delays in administrative procedures have direct consequences on housing conditions, access to work, and dependence on the welfare system. They reinforce barriers to healthcare access such as self-stigma and shame, the intensity of psychopathology and trust in others, and geographical distance. These barriers, in turn, exacerbate the corresponding risk factors (**Figure 1**). Another example of known barriers to accessing care is the lack of screening tools tailored to the specific population of RAS, which is linked to a lack of training in cultural competence among healthcare professionals and their ability to deal with issues related to psychological trauma, a lack of specific therapeutic approaches, and ultimately inadequate health insurance coverage. These barriers exacerbate the severity of psychopathology, cumulative stress, and social isolation, as well as living conditions and access to work. These well-documented risk factors in turn reinforce the corresponding barriers to care (**Figure 2**).

## 5 Conclusion

Consequently, we believe that adopting a broader perspective on the experiences of RAS in their relationships with society and healthcare would enable the design of healthcare services that are better suited to their needs, for example by increasing the mobility of healthcare professionals and more systematically combining legal resources with therapeutic proposals, or by enhancing and promoting social workers’ action through financial and logistical ressources, as we know how social integration is predominant predictivie factor of positive evolution. Moreover, we believe that immigration procedures should take into account in their own process the iatrogenic effects of home office interviews and the complex relationship between trauma (mostly resulting from sexual violence) and the ability to reveal personal informations.

A global holistic approach that proposes an understanding of the complex interactions between post-migration risk factors and personal or structural barriers to access to mental health care should be promoted in order to increase the cultural acceptability of we are able to offer.

This broader perspective could also encourage researchers to develop more comprehensive concepts of the experience of RAS after their arrival in high-income countries and enable the development of therapeutic offers specifically directed toward the complex mental health needs of RAS.
